# Decreasing High Failure Rate of Vaccinations in Patients With Chronic Kidney Disease; not Just a Matter of Quantity

**DOI:** 10.5812/hepatmon.7110

**Published:** 2012-07-30

**Authors:** Theodoros Eleftheriadis, Vassilios Liakopoulos, Ioannis Stefanidis

**Affiliations:** 1 Nephrology Department, Medical School, University of Thessaly, Larissa, Greece

**Keywords:** Vaccination, Hepatitis B, Kidney Failure, Chronic

Dear Editor,

In their recent interesting published manuscript, Ahmadi et al. showed that in predialysis patients with chronic renal failure (CRF) doubling the dose of Hepatitis B virus (HBV) vaccine from 20mcg to 40mcg was not benefit regarding the seroconvertion rate [1]. Although somewhat provocative, the above highlights the complexity of the immune system and the difficulties in interfering effectively and safely with its function.

In CRF patients the impaired immune function is multifactorial. The impaired interaction between antigen presenting cells (APCs) and T-cells is indicated by the decreased T-cell proliferation and ζ-chain phosphorylation after a major histocompatibility complex (MHC) dependent T-cell receptor (TCR) stimulation and certainly contributes to the impaired immune function. Decreased MHC and TCR expression, altered expression of the adhesion molecules lymphocyte function antigen-1 (LFA-1) and intercellular adhesion molecule-1 (ICAM-1), as well as reduced expression of the co-receptors CD80/CD86 in APCs and CD28 in T-cells may be responsible for it. Besides the impaired APC-T-cell interaction; other mechanisms have been also proposed for the acquired immunity disturbances in CRF patients. Increased soluble CD40 molecules, which inhibit the interaction between T-cells and B-cells, decreased TCR complex ζ-chain expression, altered cytokine status, which affects T-cell differentiation and finally increased apoptosis of T-cells and B-cells have all been incriminated for the impaired adaptive immune response [2]. Recently, our group showed that the immunomodulatory enzyme indoleamine 2, 3-dioxygenase is increased in CRF patients and negatively affects the immune response to HBV vaccine [3]. The above complexity indicates that the hypo responsiveness to HBV vaccine, which is observed in CRF patients, cannot be overcome simply by increasing the dose of the antigen.

The solution could be in the development of new vaccines with adjuvants other than the aluminum hydroxide, which is generally used. For example, an approved vaccine against HBV for CRF patients contains mono phosphoryl lipid A (MPL). MPL is a low-toxicity derivative of lipopolysaccharide that interacts with Toll-like receptors and the mentioned vaccine showed improved immunogenicity when administered in CRF patients [4]. Recent advances in immunology revealed that early innate immune signals shape subsequent adaptive responses and this has led to the design and development of more specific and focused adjuvants [5]. Certainly, safe and more effective vaccines will be available soon for CRF patients, which are characterized by impaired adaptive immune response.
